# The seminal plasma proteome dataset of the giant panda

**DOI:** 10.1016/j.dib.2025.112419

**Published:** 2025-12-24

**Authors:** Shili Zhou, Tao Wang, Jiasong Chen, Feiping Li, Shenfei Wang, Xianbiao Hu, Mingyue Zhang, Rong Hou, Yuliang Liu, Kailai Cai

**Affiliations:** a School of Pharmacy, Chengdu University, Chengdu 610106, China; b School of Basic Medical Sciences, Chengdu University, Chengdu 610106, China; c Chengdu Research Base of Giant Panda Breeding, Chengdu 610081 Sichuan, China; d The Conservation of Endangered Wildlife Key Laboratory of Sichuan Province, Chengdu 610081 Sichuan, China

**Keywords:** Giant panda, Seminal plasma, Proteome, WD40 repeats proteins

## Abstract

For the ex-situ conservation of giant pandas, both collecting and preserving semen are important methods. The seminal plasma is rich in nutrients and bioactive substances, such as proteins, carbohydrates, lipids, amino acids, and hormones, which play an important role in the reproduction and reproductive health of the species. Understanding the composition and function of proteins in the giant panda seminal plasma proteome can provide valuable insights into their reproductive biology and help develop strategies to improve their reproductive success in captivity, which is essential for giant panda conservation. This is the first study to analyze the seminal plasma proteins of giant pandas through proteomics, identifying 1125 proteins. These proteins are related to protein turnover, translation, and metabolism. The seminal plasma proteins of giant pandas were then compared to those of humans, pigs, and sheep, and many unique proteins were found in giant panda samples. Among these proteins, the WD40 repeat-containing proteins have been identified and implicated in sperm function and fertility.

Specifications Table

**Table:** 

Subject	Biology
Specific subject area	Understanding of seminal plasma proteome of giant panda*.*
Type of data	Table, Figure, Raw, Analyzed
Data collection	All the samples required for the data were collected during artificial insemination and cryogenically stored at the Sichuan Key Laboratory of Conservation Biology for Endangered Wildlife (Chengdu Research Base of Giant Panda Breeding) following standard routine procedures. Through protein extraction, shotgun proteomics analysis was performed using the EASY-nLCTM 1200 UHPLC system (Thermo Fisher) coupled with the Orbitrap Q Exactive HF-X mass spectrometer (Thermo Fisher) operating in the data-dependent acquisition (DDA) mode*.*
Data source location	Country: Sichuan, China.
Data accessibility	Repository name: ProteomeXchange Consortium Data identification number: IPX0006858001 Direct URL to data: http://proteomecentral.proteomexchange.org
Related research article	No

## 1 Value of the Data

• The giant panda (Ailuropoda melanoleuca) is not only a flagship species for global biodiversity conservation, but also serves as a political and diplomatic ambassador for China, and a cultural icon. In recent years, both in-situ and ex-situ conservation measures have been implemented to different degrees of success, however, the species is still vulnerable to extinction.
• For the captive population, the low proportion of naturally mating male giant pandas is a major limiting factor for their reproductive efficiency, utilization, and genetic diversity. With artificial insemination techniques, this problem can be overcome and genetic management can also be facilitated, making the preservation and efficient use of individual giant panda semen significant for maintaining the entire captive population.
• Seminal plasma proteins play an important role in sperm function, and many of the tissue-specific proteins can accurately indicate the pathological processes of the tissues from which they originate. Therefore, identifying and analyzing the whole proteomics of giant panda seminal plasma using a gel-free and label-free shotgun proteomics approach can help us better understand its reproductive health status and reproductive capacity.
• On the other hand, identifying some biomarkers associated with male fertility, sperm quality, and reproductive success can be used to develop diagnostic tools for evaluating the male reproductive health of giant pandas, thus enabling better conservation of giant pandas.

## 2 Background

The giant panda (Ailuropoda melanoleuca) is a unique and vulnerable species endemic to China. Conserving the giant panda is of particular ecological, social, political and economic significance.

Seminal plasma is essential for successful implantation and pregnancy [1,2]. The seminal plasma proteome contains thousands of proteins and includes many tissue-specific proteins that might accurately indicate a pathological process in the tissue of origin [3]. Specific proteins present in seminal plasma may be used as discriminant variables with the potential to predict sperm motility and fertility [4].

Recently, Martins, A.D. et al. elucidated the potential role of differentially expressed proteins in the seminal plasma as a diagnostic biomarker for primary and secondary infertility [5]. The correlation between semen protein composition, sperm activity and fertility in animals such as cattle [6], goats [7], pigs [8] and chicken [9] has been explored. Zhu et al. quantified 35 metabolome molecules with distinct age-related trends in the giant panda seminal plasma [10]. The study aimed to identify the whole proteome profiles of giant panda seminal plasma using a gel-free, label-free shotgun proteomics approach, it is possible to better understand its reproductive health status and reproductive capacity, and search for relevant biomarkers for evaluating male reproductive health.

## 3 Data Description

### 3.1 Protein identification in giant panda

**Fig. 1:**
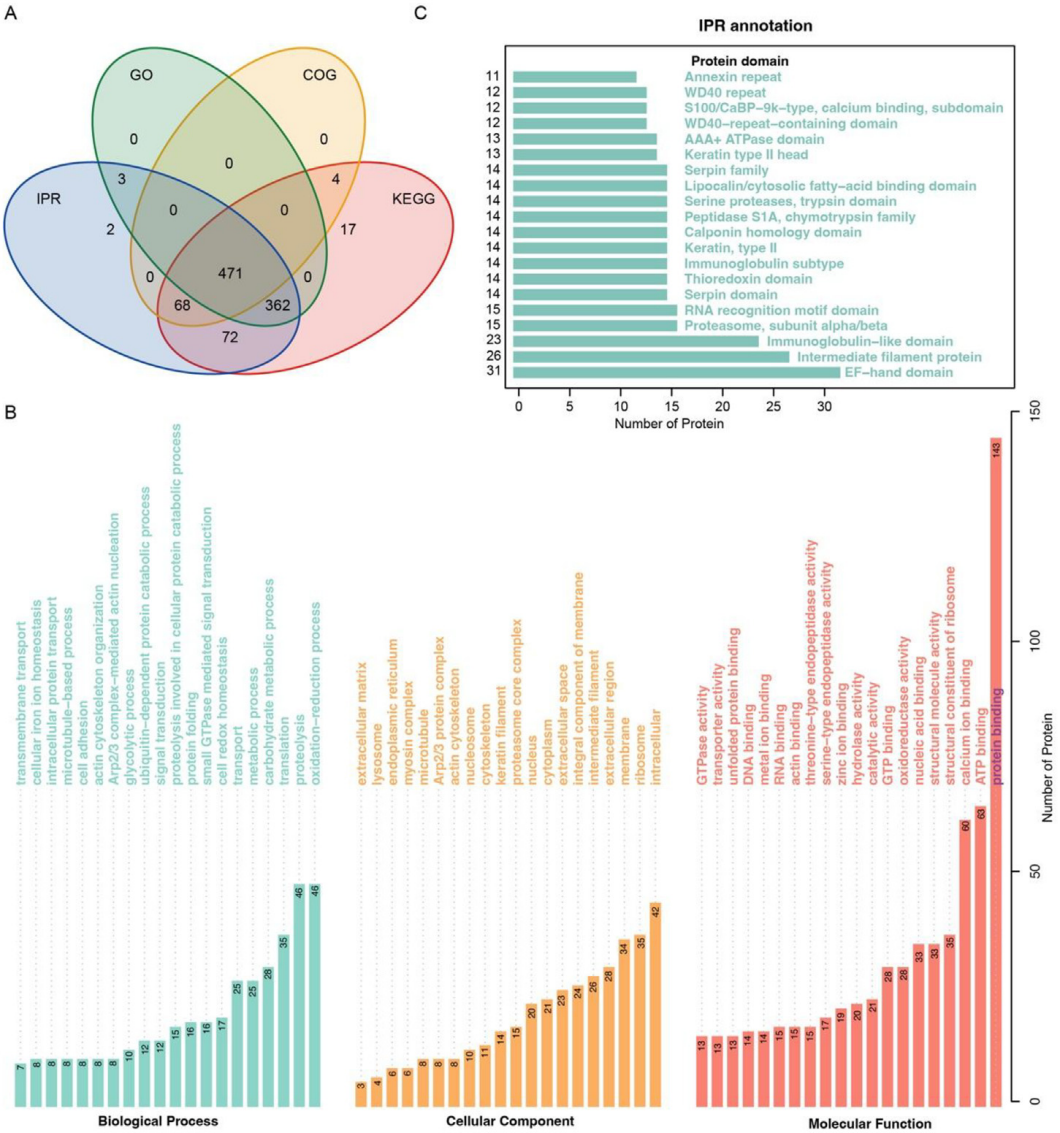
The Venn diagram displayed the annotation results of the identified proteins in four databases (A), B and C showing the GO and IPR annotations, respectively. The bar represents the number of proteins.

**Fig. 2:**
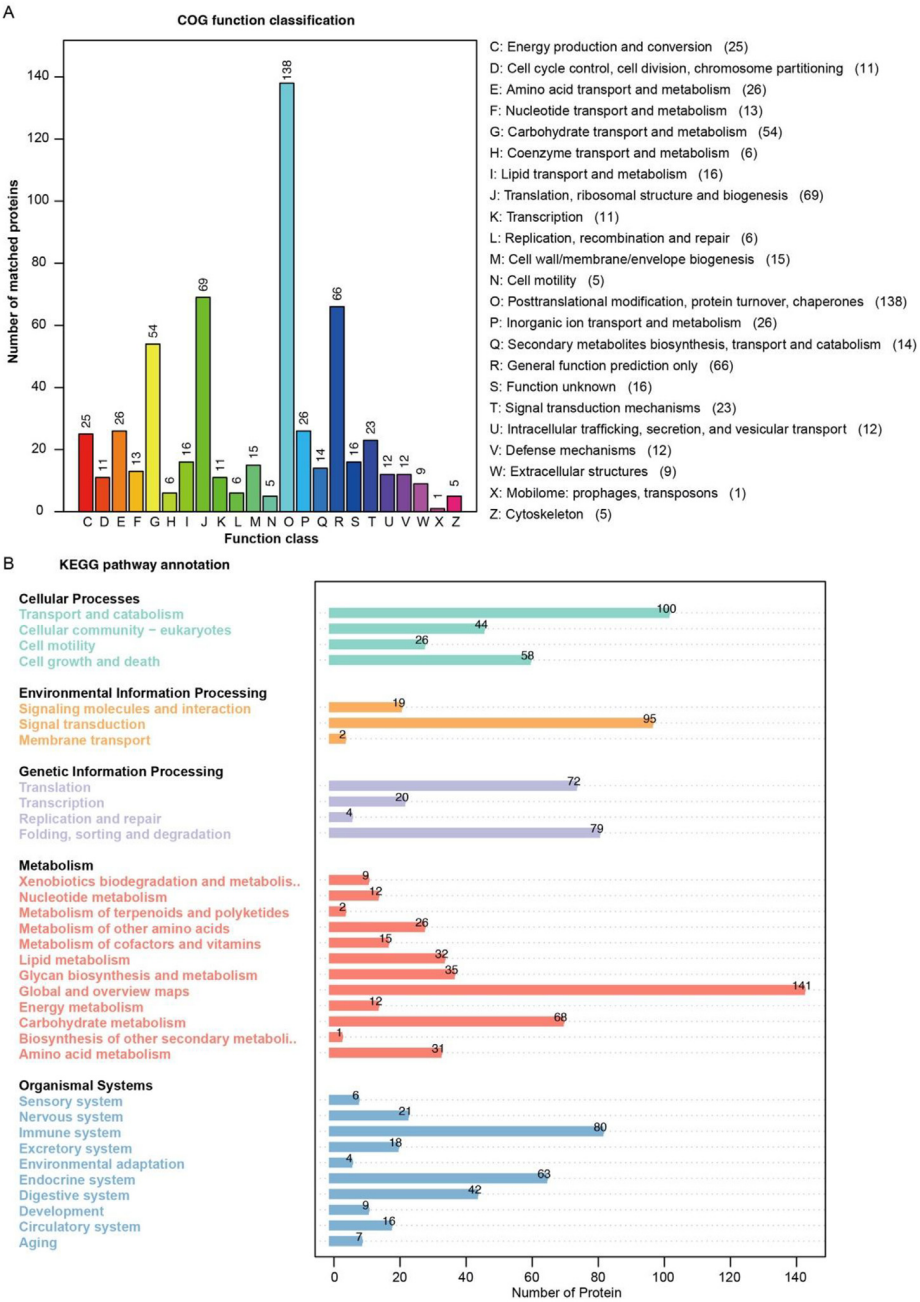
The COG (A)and KEGG (B) annotation of detected seminal plasma proteins. The bar represents the number of proteins.

A total of 1125 proteins in the seminal plasma (Table S1, Supporting Information) were identified using the label-free shotgun proteomic approach. In this study, we used four databases (GO, IPR, KEGG and COG) for annotation, most proteins could be annotated (Fig. 1A), KEGG annotated the most proteins in multiple databases, accounting for 88.36 % (994/1125, the proportion of proteins successfully annotated by the corresponding database relative to the total number of proteins subjected to annotation), and GO, IPR, and COG annotated 74.31 %, 86.93 %, and 48.27 %, respectively. Based on the results of the GO annotation analysis of the cellular compartment, there is a broad range of sources for seminal plasma proteins, and the primary sources are intracellular, ribosome and membrane (Fig. 1B). The molecular function of these proteins is associated with protein binding, ATP binding, and calcium ion binding; the biological processes of these proteins are involved in the oxidation–reduction process, proteolysis, translation, and carbohydrate metabolic process (Fig. 1B). We found that these proteins are rich in EF-hand domain, intermediate filament protein, and immunoglobulin-like domain (Fig. 1C), which should match their common functions in GO annotation. Based on COG and KEGG annotation, it also highlights that these proteins are involved in protein turnover, translation, and metabolism (Fig. 2A, Fig. 2B).

### 3.2 Comparison of the seminal plasma proteins in giant pandas and other species

**Fig. 3:**
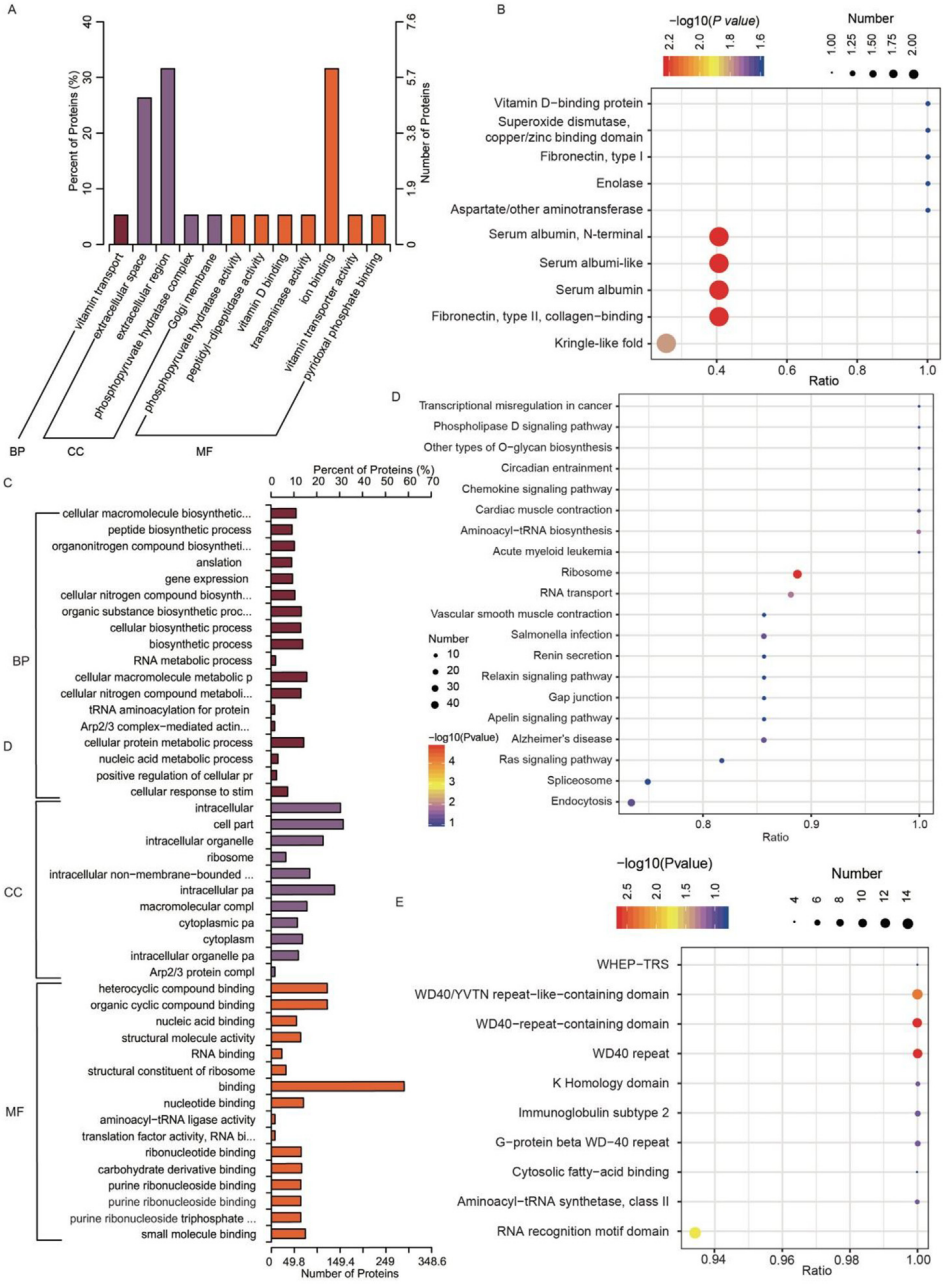
The GO (A) and KEGG (B) annotation of shared seminal plasma proteins between panda and three other species (human, pig, sheep). (C) and (D) represent the GO and KEGG annotation of panda-specific seminal plasma proteins compared to three other species, The results of IPR analysis have also been presented simultaneously (E).

A comparison of the seminal plasma proteins identified in giant pandas and three other species (human, pig, sheep, Table S2) revealed that pandas share 25 genes (corresponding to 25 proteins) with the other species (Table S3), and have 598 unique genes (corresponding to 598 proteins, Table S4). We also merge the UniProt amino acid sequence libraries of the three species (human, pig, and sheep) and BLAST with the identified panda seminal proteins to find similar proteins. Finally, it was determined that 998 proteins could be used as a homologous protein list, and the 598 panda-specific seminal plasma proteins were included in this list. Subsequent functional enrichment analysis shows that 25 shared proteins mainly play a role in the ion binding process (Fig. 3A). The COG annotated these proteins in serum albumin and fibronectin. Human serum albumin (HSA) is the most abundant seminal plasma protein and an important constituent of seminal plasma (Fig. 3B).

In the comparative analysis, most proteins were unique to giant pandas. Functional analysis was also conducted on panda unique proteins, these proteins were enriched in the ribosome pathway (Fig. 3C), and were also related to binding function, including heterocyclic compound binding and nucleic acid binding (Fig. 3D).

### 3.3 Panda unique proteins

Additionally, these panda unique proteins were enriched in WD40 repeat and WD40 repeat-containing domain items (Fig. 3E). The WD40 repeat is a short motif structure consisting of approximately 40 amino acids often in a tryptophan-aspartic acid (W-D) dipeptide [11]. The WD40 domain usually comprises several repeats and is found in many proteins involved in various cellular processes [12].

In this study, twelve WD40 repeat-containing proteins were identified, and we presented fundamental information about these repeats (Table 1). Through a literature review, we discovered PAFAH1B1 plays important roles in spermatogenesis, fertilization and subsequent embryonic development in mice [13]. A key point of interest is that WD40 repeat-containing proteins, typically found in the cytoplasm, appear in more significant quantities within seminal plasma. Additionally, exosome-mediated transport could also play a significant role.

**Table 1:** Summary of WD40 repeat-containing proteins in seminal plasma.

Uniprot ID	Protein name	Gene symbol	Molecular function
G1LGH1	WD repeat domain 1	*WDR1*	regulation of actin cytoskeleton
G1KZF6	Receptor for activated C kinase 1	*RACK1*	regulation of protein kinase C activity
G1LLT4	Platelet-activating factor acetylhydrolase IB subunit alpha	*PAFAH1B1*	regulation of microtubule cytoskeleton
G1LGH6	Coatomer subunit beta	*COPB2*	a subunit of the Golgi coatomer complex
D2HI58	Uncharacterized protein	unkown	unkown
G1LI97	Actin-related protein 2/3 complex subunit	*ARPC1A*	regulation of actin filament polymerization
G1LI94	unkown	unkown	unkown
D2HX06	Uncharacterized protein	unkown	unkown
G1LEQ8	SEC31 homolog A, COPII coat complex component	*SEC31A*	anterograde transport from the endoplasmic reticulum (ER) to the Golgi apparatus
D2HX51	Coronin	unkown	unkown
G1LEX2	EMAP like 4	*EML4*	microtubule stabilization, intracellular transport, and cell signaling
G1M6T5	Coatomer subunit alpha	*COPA*	retrograde transport from the Golgi to the endoplasmic reticulum (ER).

## 4 Experimental Design, Materials and Methods

### 4.1 Sample collection

Semen from four sexually mature giant pandas (aged between 9 and 16 years, the average was 11.5 ± 3.32) was collected by electroejaculation during the breeding season according to the previous methodology [14]. Semen was collected into a plastic container and immediately placed in a centrifuge. An aliquot of 0.5 mL of fresh semen was centrifuged at 900 × *g* for 30 min at 4 °C to separate seminal plasma from spermatozoa. Seminal plasma was transported at 4 °C and frozen at −80 °C until further use. All samples were collected during artificial insemination and cryogenically stored following a standard, routine procedure at the Sichuan Key Laboratory of Conservation Biology for Endangered Wildlife, Chengdu Research Base of Giant Panda Breeding.

Due to the challenges in obtaining sufficient semen for analysis, samples from four giant pandas are combined to create a biological replicate for proteomics. The semen sample was initially sonicated three times in ice and lysed in lysis buffer containing 100 mM NH4HCO3 (pH 8), 6 M Urea, and 0.2 % SDS (w/v), followed by 5 min ultrasonication on ice. The lysate was centrifuged at 12,000 *g* for 15 min at 4 °C and the supernatant was transferred to a clean tube.

### 4.2 Protein extraction

Extracts from the sample were reduced with 2 mM DTT for 1 h at 56 °C and alkylated with sufficient Iodoacetamide for 1 h at room temperature in the dark. Then 4 times the volume of precooled acetone was mixed with samples by well vortexing and incubated at −20 °C for at least 2 h. The sample was then centrifuged, and the precipitation was collected. After washing twice with cold acetone, the pellet was dissolved by a dissolution buffer containing 0.1 M triethylammonium bicarbonate (TEAB, pH 8.5) and 6 M urea. Protein concentration was determined again by Bradford protein assay. The supernatant from the sample, containing precisely 0.12 mg of protein was digested with Trypsin Gold (Promega) at 1:50 enzyme-to-substrate ratio. After 16 h of digestion at 37 °C, peptides were desalted with a C18 cartridge to remove the high urea, and desalted peptides were dried by vacuum centrifugation.

### 4.3 Shotgun proteomics detection and analyses

Shotgun proteomics analyses were performed using an EASY-nLCTM 1200 UHPLC system (Thermo Fisher) coupled with an Orbitrap Q Exactive HF-X mass spectrometer (Thermo Fisher) operating in the data-dependent acquisition (DDA) mode. A sample volume containing 2 μg of total peptides was injected onto a home-made C18 Nano-Trap column (2 cm × 100 μm, 3 μm). Peptides were separated on a home-made analytical column (15 cm × 150 μm, 1.9 μm), using a 60 min linear gradient from 5 to 100 % (v/v) eluent B (0.1 % (v/v) FA in 80 % (v/v) ACN) in eluent A (0.1 % (v/v) FA in H_2_O) at a flow rate of 600 nL/min. The detailed solvent gradient is listed as follows: 5–10 % (v/v) B, 2 min; 10–30 % (v/v) B, 49 min; 30–50 % (v/v) B, 2 min; 50–90 % (v/v) B, 2 min; 90–100 % (v/v) B, 5 min. Q-Exactive HF-X mass spectrometer was operated in positive polarity mode with a spray voltage of 2.3 kV and capillary temperature of 320 °C. Full MS scans ranging from 350 to 1500 *m/z* were acquired at a resolution of 60,000 (at 200 *m/z*) with an automatic gain control (AGC) target value of 3 × 10^6^ and a maximum ion injection time of 20 ms. The 40 most abundant precursor ions from a full MS scan were selected for fragmentation using higher energy collisional dissociation (HCD) fragment analysis at a resolution of 15,000 (at 200 *m/z*) with an AGC target value of 1 × 10^5^, a maximum ion injection time of 45 ms, a normalized collision energy of 28 % ( % NCE, normalized collision energy), an intensity threshold of 2.2-e4, and the dynamic exclusion parameter of 20 s.

The resulting spectra from each fraction were searched separately against ‘P101SC18111984–01-ailuropoda_melanoleuca.fasta’ by the search engines: Proteome Discoverer 2.2 (PD 2.2, thermo). The searched parameters were as follows, a mass tolerance of 10 ppm for precursor ion scans and a mass tolerance of 0.02 Da for the product ion scans were used, carbamidomethyl was specified in PD 2.2 as fixed modifications, oxidation of methionine (M) and acetylation of the N-terminus were specified in PD 2.2 as variable modifications and a maximum of 2 miscleavage sites were allowed.

For protein identification, a protein with at least one unique peptide was identified at FDR <1.0 % (%, False Discovery Rate) on peptide and protein levels, respectively. Proteins containing similar peptides that could not be distinguished based on MS/MS analysis were grouped separately as protein groups. Precursor quantification based on intensity was used for label-free quantification. Firstly, the relative quantification value of each PSM (Peptide Spectrum Matches) in each sample can be obtained according to the peak area of the original lower machine, and then the relative quantification value of the unique peptide is corrected according to the quantitative information of all PSMs contained in the identified unique peptide, and then the relative quantification value of each protein is corrected according to the quantitative information of all unique peptides contained in each protein. Gene Ontology (GO) and InterPro (IPR) analysis were conducted using the InterProScan-5 program against the non-redundant protein database (including Pfam, PRINTS, ProDom, SMART, ProSiteProfiles, PANTHER) [15]. The databases COG (Clusters of Orthologous Groups) and KEGG (Kyoto Encyclopedia of Genes and Genomes) were used to analyze the protein family and pathway, we first aligned the identified proteins to the above database by BLAST (blastp, *e-value* ≤ 1e-4), the selected the alignment results with the highest score for annotation, based on the alignment results, assign each protein to a specific COG or KEGG category.

## Limitations

A limitation of our research is that Sample collection is extremely difficult. Giant pandas are small in number and have a relatively sensitive personality, and it is difficult to obtain sufficient and representative semen samples, which may lead to insufficient proteomic data sets. By establishing a dataset of the giant panda seminal plasma proteome and identifying specific proteins and biomarkers associated with reproduction, researchers may be able to develop new diagnostic and therapeutic approaches to improve male panda reproductive health.

## Ethics Statement

We confirm that our research strictly adheres to the guidelines for authors provided by Data in Brief in terms of ethical considerations.

## Credit Author Statement

**Shili Zhou:** Analyzed the data, Contributed to writing the manuscript. **Tao Wang:** Analyzed the data, Contributed to writing the manuscript. **Kailai Cai**: Conceived and designed the experiments, Performed the experiments, Contributed to writing the manuscript. **Yuliang Liu**: Conceived and designed the experiments. **Rou Hou**: Conceived and designed the experiments. **Jiasong Chen:** Performed the experiments. **Feiping Li:** Performed the experiments. **Shenfei Wang:** Performed the experiments. **Xianbiao Hu:** Performed the experiments. **Mingyue Zhang:** Performed the experiments.

## Data Availability

iProX partner repositoryIPX0006858001 (Original data)

## Appendix Supplementary materials

Image, application 1
